# The Use of Hyaluronic Acid in the Non-Surgical Treatment of Periodontitis—An Umbrella Review

**DOI:** 10.3390/biomedicines13040998

**Published:** 2025-04-20

**Authors:** Wojciech Niemczyk, Jacek Matys, Rafał Wiench, Jacek Żurek, Marzena Dominiak

**Affiliations:** 1 Medical Center of Innovation, Wroclaw Medical University, Krakowska 26, 50-425 Wroclaw, Poland; 2 Department of Dental Surgery Medical, University of Wroclaw, Krakowska 26, 50-425 Wroclaw, Poland; marzena.dominiak@umw.edu.pl; 3 Department of Periodontal Diseases and Oral Mucosa Diseases, Faculty of Medical Sciences in Zabrze, Medical University of Silesia, Pl. Traugutta 2, 41-800 Zabrze, Poland; rwiench@sum.edu.pl; 4 Specialist Medical Practice, Polne Wzgórze 11 Street, 32-300 Olkusz, Poland

**Keywords:** hyaluronic acid, periodontitis, dental scaling, periodontal debridement, root planing

## Abstract

**Background**: Periodontitis is a prevalent inflammatory condition that destroys periodontal tissues. Scaling and root planing (SRP) is the gold standard for non-surgical treatment; however, its efficacy may be limited in cases with complex dental issues. This umbrella review aims to evaluate the effectiveness of hyaluronic acid (HA) as an adjunct to scaling and root planing (SRP) in enhancing clinical outcomes for periodontitis management. **Methods**: A comprehensive review of five systematic reviews, including meta-analyses where available, was conducted to synthesize evidence on the adjunctive use of HA with SRP. The studies were evaluated using the AMSTAR-2 quality assessment tool to determine methodological rigor. Data on clinical parameters such as probing depth (PD), clinical attachment level (CAL), bleeding on probing (BOP), gingival index (GI), and plaque index (PI) were extracted and analyzed. **Results**: The findings indicate that HA supplementation leads to moderate improvements in PD, CAL, BOP, GI, and PI compared to SRP alone. Notable reductions in PD and gains in CAL were observed, with some meta-analyses showing statistically significant benefits. However, the heterogeneity in HA concentrations (0.2–1.4%), application methods, treatment frequencies, and follow-up durations (1 week to 12 months) limits definitive conclusions. Additionally, HA did not significantly affect the reduction in *P. gingivalis* prevalence. **Conclusions**: The use of HA in conjunction with SRP shows promise in enhancing the efficacy of non-surgical periodontal therapy. However, the heterogeneity in the quality and methodologies of the studies indicates the necessity for high-quality, standardized randomized controlled trials to establish clear clinical guidelines for the application of HA in the treatment of periodontitis.

## 1. Introduction

Periodontitis is an inflammatory condition that results in the gradual destruction of the supportive tissues of the teeth. The host inflammatory response, initiated by bacteria, compounds, or other causes of periodontal diseases, including diabetes and smoking, among others, is the primary mechanism responsible for tissue destruction [1,2,3,4]. Nevertheless, the progression of periodontitis is also closely associated with its specific biotype [5]. Periodontitis represents a significant public health concern, with the potential to result in a range of adverse outcomes, including tooth loss and the exacerbation of other chronic diseases [6,7,8,9,10]. The condition frequently manifests as gingivitis, which is typified by bleeding, edema, and discomfort. If left unaddressed, it can advance to periodontitis, a condition marked by the deterioration of periodontal attachment and supporting bone [11,12]. It is widely accepted that scaling and root planing (SRP), a non-surgical treatment for periodontitis, represents the gold standard for this indication. The efficacy of this approach has been well documented in several systematic reviews [13,14,15,16].

In light of the potential limitations of SRP in certain cases, due to anatomical constraints or increased pocket depth, the use of systemic and topical coadjuvants may be warranted in select instances [17,18]. Topical therapies ensure the optimal delivery of high concentrations of pharmacologic agents to periodontal tissues [19]. Adjuvant therapies can be either systemic or topical. However, the systemic administration of these drugs necessitates the use of high doses to achieve optimal concentration at the targeted site. Additionally, patient compliance with the prescribed administration regimen is a crucial factor. Furthermore, these drugs may potentially induce adverse effects. Consequently, several local treatments, including gels, fibers, and chips loaded with a variety of active molecules or drugs, have been developed and evaluated in clinical settings. This underscores the critical importance of utilizing specialized carriers for the effective delivery of pharmaceutical agents [20]. Moreover, the utilization of topical agents during non-surgical periodontal therapy to disaggregate biofilms is a promising approach [21]. The principal advantages of these treatments are the targeted delivery of active pharmaceutical agents to the precise site of the lesions, the reduced risk of adverse effects, and the potential for three-dimensional stabilization of the blood clot [22,23]. Hyaluronic acid (HA) is a glycosaminoglycan that belongs to the high-molecular-weight polysaccharide class and is commonly found in bodily fluids such as gingival crevicular fluid (GCF), saliva, serum, and synovial fluid. Furthermore, hyaluronic acid represents a significant component of both mineralized and non-mineralized extracellular matrices [24,25]. Native HA is a biomolecule that is present in oral tissues, including the gingiva and periodontal ligaments, as well as in low quantities in cementum and the alveolar bone [26]. HA is available in two forms. Low molecular weight HA (LMW-HA) has been shown to promote angiogenesis, while high molecular weight (HMW-HA) has an opposite effect. The LMW-HA plays a role in signaling tissue damage and mobilizing immune cells. Conversely, the HMW-HA exerts a suppressive effect on the immune response, thereby mitigating the risk of exacerbated inflammation [27,28].

The production of HA is regulated by fibroblast growth factor 2 (FGF2), a protein that is expressed in human periodontal ligament cells. The anti-inflammatory, anti-edematous, and anti-bacterial activities of HA have been investigated in the context of dentistry, with particular emphasis placed on the use of this material in the treatment of gingival disorders resulting from the presence of microorganisms within subgingival plaques [29]. HA plays a regulatory role in the inflammatory and immune response, functioning as an antioxidant. Among the most significant attributes of HA is its capacity for regeneration, which is attributable to its osteoinductive effect [30,31]. HA has been demonstrated to promote the migration of endothelial cells, which form a network for the deposition of bone tissue. Additionally, this mucopolysaccharide has been shown to transport proteins that act as growth factors, including bone morphogenetic protein-2 and osteopontin. Additionally, HA has been demonstrated to possess anti-inflammatory and immunosuppressive properties, which are mediated by its ability to form a complex that inhibits proteases [30,32,33,34].

This umbrella review aims to evaluate the effectiveness of HA as an adjunct to SRP in the treatment of periodontitis by synthesizing evidence from systematic reviews. Specifically, the review seeks to determine whether the addition of HA to SRP results in superior clinical outcomes compared to SRP alone. Key clinical parameters assessed include probing depth (PD), clinical attachment level (CAL), bleeding on probing (BOP), gingival index (GI), and plaque index (PI). Furthermore, the review will examine the impact of HA on bacterial reduction, with a particular focus on its effect on *Porphyromonas gingivalis*, a key pathogen in periodontal disease. By systematically analyzing existing evidence, this review aims to provide clarity on the therapeutic value of HA and identify gaps for future research.

## 2. Materials and Methods

In accordance with the PRISMA guidelines, the protocol for this systematic umbrella review was registered in the International Prospective Register of Systematic Reviews (PROSPERO) on 24 November 2024 (PROSPERO 2024CRD42024614053).

### 2.1. Focused Question

This review was conducted using the PICO framework [35] as follows: in patients with chronic periodontitis (Population), does the adjunctive use of hyaluronic acid in conjunction with SRP (Intervention) result in a more efficacious improvement of clinical parameters (Outcome) compared to SRP alone (Comparison)?

### 2.2. Search Strategy

The structure of the review adhered to the guidelines proposed by Cant et al. in 2022 [36]. A review of the literature was conducted using electronic databases up to 21 October 2024. The databases included MEDLINE (PubMed), Google Scholar, Embase, and Scopus. The syntaxes employed for each database, along with the restrictions and the number of results obtained, are presented in Table 1. Furthermore, the article collection was conducted using the snowball method, which involved searching for forward and backward citations. Among the inclusion criteria were systematic reviews with or without a meta-analysis. Furthermore, the articles had to be accessible in full and written in English in order to be included. The databases were systematically searched by three authors, who were independent from one another. The authors used the same search syntax and restrictions.

### 2.3. Study Selection

The objective of this systematic review was to assess the potential utility of hyaluronic acid as an adjunctive treatment for scaling and root planing. It was hypothesized that the use of hyaluronic acid during SRP would effectively support the therapies by further improving clinical parameters. The criteria for the inclusion of articles in and the exclusion of articles from this review are presented in Table 2.

### 2.4. Quality Assessment

Two independent reviewers, (W.N.) and (R.W.), were responsible for conducting quality assessments of the included studies. The methodological quality of the studies was evaluated in accordance with the AMSTAR-2 guidelines [37,38]. Each of the systematic reviews analyzed was subject to evaluation of 16 parameters, of which 7 were critical (Q2, Q4, Q7, Q9, Q11, Q13, Q15). The evaluation of all parameters is shown in Table 3. Each systematic review was then assigned a score according to the guidelines in Table 4.

### 2.5. Data Extraction

In order to extract relevant data, the two authors (W.N.) and (R.W.) jointly determined the desired data from each of the included articles and then independently searched the articles to find relevant data. Cohen’s K test was utilized as a methodology to quantify the level of inter-reviewer agreement [44]. In addition to seeking responses to the questions posed by AMSTAR-2, the authors endeavored to identify pertinent data, including the year of publication, the total number of patients included in the studies, the total number of sites involved in the studies, the mean age of the subjects, the gender distribution, the period during which the articles were searched, and the tool utilized to assess the quality of the studies. The quality of the included studies, the number of studies included in the analysis, the types of articles analyzed, the percentage concentration of HA used in studies, the number of HA application sessions, whether a meta-analysis was performed, the extent of the follow-up period, and the conclusions of the authors of the systematic reviews were also considered.

## 3. Results

**Figure 1:**
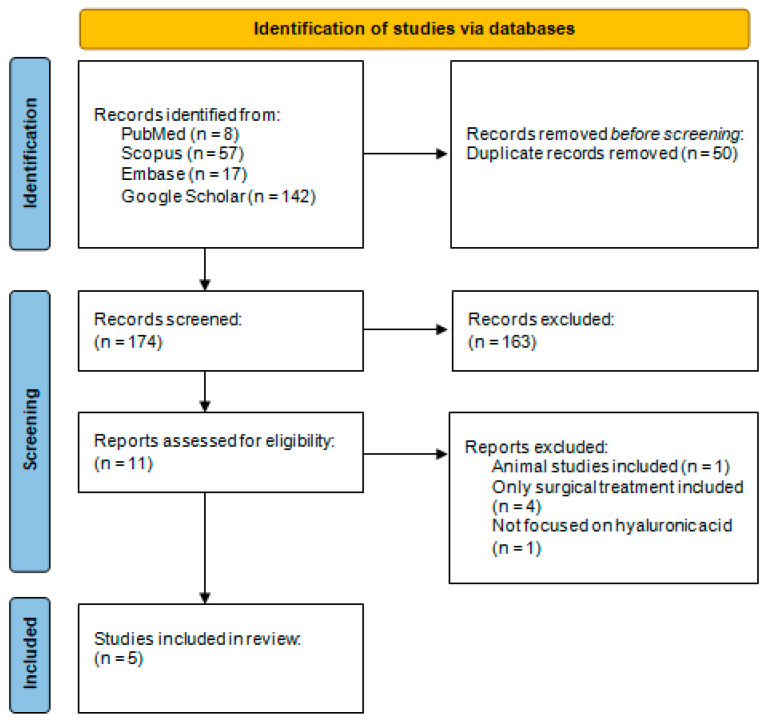
PRISMA flow chart.

Following the removal of duplicates from electronic databases, 174 articles were identified for further processing. Of these, 11 reports were assessed for eligibility, with one being rejected due to the inclusion of animal studies [45] and four being rejected due to the inclusion of studies on the surgical treatment of periodontal disease [46,47,48,49]. Additionally, one of the systematic reviews addressed gels in the non-surgical treatment of periodontitis without focusing on hyaluronic acid [22]. All the data mentioned above are included in the Figure 1.

### 3.1. Characteristics of the Included Systematic Reviews

All five systematic reviews were published from 2019 to 2024. The total number of patients included in the studies ranged from 113 to 579, and none of the articles provided a comprehensive account of the total number of sites that were investigated. Only one of the reviews provided data on the mean age and gender distribution [39]. The review by Eliezer et al. [41] was the sole review to omit the search period for articles included in the review, while Alshehri and Alharbi [39] were the only authors to fail to report the tool used to assess the quality of the included papers. Table 5 presents the characteristics of the included studies.

### 3.2. Synthesis of Results

Of the five included articles, three performed a meta-analysis [39,40,41]. The follow-up time ranged from 1 week to 1 year. The concentrations of hyaluronic acid used in the included studies generally ranged from 0.2% to 0.8%; however, one review (Alshehri and Alharbi, 2023) [39] reported concentrations up to 1.4%. The number of acid application sessions also varied widely between studies, ranging from 1 to 29, when patients also applied acid at home twice a day for 2 weeks. Of the five systematic reviews, three included only randomized controlled trials [41,42,43], one included both randomized and non-randomized control trials [39], and one included both prospective and retrospective studies in the analysis [40]. A summary of the key points from each article is presented in Table 6.

Alshehri and Alharbi were the only authors to perform a systematic review on microbiological parameters, namely, the effect of HA in SRP on *Porphyromonas gingivalis* reductions. By performing a data synthesis using meta-analysis, they showed that the additional use of HA does not improve this parameter. This was also the only review in which the concentration of HA ranged from 0.2 to 1.4%. In all the rest, the concentration range was between 0.2 and 0.8%. The rest of the authors performed four systematic reviews focused on clinical parameters. All of them showed a positive effect on their improvement, both in reviews using narrative data analysis and those using meta-analysis. Among the clinical parameters analyzed by the authors that improved were plaque index, bleeding on probing, pocket depth, clinical attachment level, and the gingival index. Unfortunately, as many as two of these four reviews did not extract data regarding the number of sessions of HA application. In the other two, the number of sessions ranged from 1 to 7.

#### 3.2.1. Probing Depth (PD)

In a meta-analysis, Dharmadhikari showed that the average reduction in probing depth was 0.79 times greater in the HA group [40]. In contrast, the meta-analysis conducted by Eliezer et al. demonstrated that the use of hyaluronic acid as an adjunctive therapy resulted in a mean reduction in PD of 0.36 mm [41]. In a narrative analysis of a systematic review, Karakostas et al. also inferred that HA improves the clinical parameter, that is, PD [42]. Rodrigues et al. reached similar conclusions [43].

#### 3.2.2. Clinical Attachment Level (CAL)

In a meta-analysis, Dharmadhikari demonstrated that the mean attachment level in the HA group was, on average, 0.31 times greater than that observed in the control group [40]. In their meta-analysis, Eliezer et al. demonstrated that the CAL gain (mean 0.73 mm) was superior to that achieved through conventional scaling and root planning [41]. Karakostas et al. highlighted that clinical attachment level values were enhanced when assessed three months post-treatment and when the trials were funded [42]. Rodrigues et al. also reported comparable outcomes in their narrative synthesis [43].

#### 3.2.3. Gingival Index (GI)

The gingival index was only described in the review conducted by Dharmadhikari. In his statistical synthesis, he demonstrated that the mean reduction in the gingival index was 0.73 times greater in the HA group [40].

#### 3.2.4. Plaque Index (PI)

As with GI, only Dharmadhikari provided a meta-analysis of the effect of HA in addition to SRP on plaque index. The author concluded that the mean reduction in plaque index was 1.42 times greater in the HA group [40].

#### 3.2.5. Bleeding on Probing (BOP)

Dharmadhikari demonstrated that the impact of HA as an additive to SRP on the BOP parameter is markedly pronounced. The mean decrease in bleeding on probing in the HA group was 4.51 times greater than that observed in the control group [40]. Nevertheless, Eliezer et al. demonstrated that the reduction in bleeding on probing in patients undergoing the addition of hyaluronic acid was approximately 15% in comparison to patients undergoing SRP alone [41]. Additionally, an improvement in this parameter was observed in the narrative analysis conducted by Rodrigues et al. [43].

#### 3.2.6. *Porphyromonas gingivalis*

The only systematic review that focused on the effect of HA on the reduction in *P. gingivalis* bacteria was conducted by Alshehri and Alharbi. The results did not support the use of HA during non-surgical mechanical therapy to reduce the prevalence of *P. gingivalis* in subgingival biofilm (odds ratio = 0.95 and 1.11 at three and six months, consecutively) [39].

## 4. Discussion

This umbrella review synthesizes evidence from five systematic reviews that evaluate the role of HA as an adjunct to SRP in the non-surgical management of periodontitis. The results suggest that HA supplementation leads to moderate improvements in clinical parameters, including PD, CAL, BOP, GI, and PI. However, the variability in study methodologies, including differences in HA concentrations, frequency of applications, and duration of follow-up, creates challenges in drawing definitive conclusions. Additionally, HA’s use did not significantly influence the reduction in *P. gingivalis* prevalence. A comparable set of bacteriological findings was obtained in a study in which HA was administered once a week in vivo. No observable effect on the number of periodontopathogenic bacteria was detected [50].

A critical aspect of the present study is the differentiation between the application methods of HA utilized in the reviewed studies. In some trials, HA was professionally applied subgingivally during SRP sessions. In other trials, patients were instructed to apply HA topically at home. The delivery method has been demonstrated to influence the bioavailability and mechanical retention of HA in the periodontal pocket, which may result in varying clinical outcomes. Consequently, comparisons between these two approaches should be interpreted with caution. Future studies should investigate these modalities separately to better understand their respective efficacies.

Other researchers have obtained divergent results in relation to microbiological parameters [51,52]. In addition to the measurement of clinical and microbiological parameters, there are studies that evaluate inflammatory biomarkers, including neutrophil elastase and human beta-defensin 2 (hBD-2). Mallikarjun et al. (2016) revealed a decrease in neutrophil elastase (NE) levels in the experimental and control groups; however, no significant difference was identified between the two [53]. NE is a significant biomarker in periodontal disease, contributing to soft tissue damage and gingival inflammation [54,55]. The study conducted by Eick and Pfister examined the relationship between NE levels and the release of NE into tissues, intending to investigate the potential of HA to mitigate this process. The capacity of HA to impede the secretion of NE can prevent additional deterioration to the periodontal tissue, consequently accelerating the healing process in periodontal therapy. Nevertheless, this study revealed no substantial impact on neutrophil enzymes [52]. In the experimental group in the study by Al-Shammari et al. in 2018, wound healing and tissue repair processes in the periodontal area were accelerated by the administration of HA [56] as a result of increased levels of the enzyme hBD-2 [57,58,59,60]. The hBD-2 enzyme has been found to have the ability to bind to microbial membranes and form gaps in them, thereby causing a change in membrane permeability and resulting in the lysis of microbial cells [59]. The anti-microbial action of hBD-2 plays a pivotal role in host defense against microbial colonization in the mucosa and gingival environment [61,62].

The favorable effect of HA is closely associated with its anti-inflammatory properties. The present findings are consistent with other studies that have highlighted the anti-inflammatory and regenerative properties of HA [63,64]. HA has been identified as a potential agent for the restoration of periodontal integrity, owing to its intricate interactions with the extracellular matrix and its components [65]. High-molecular-weight HA has been observed to impede the proliferation of fibroblasts and lymphocytes within the epithelium of periodontal lesions. In dogs, HA functions as a scaffold, thereby promoting adhesion and the proliferation of periodontal ligament cells. It is currently under discussion as a potential scaffold for incorporating selected molecules with a view to its clinical application in periodontal tissue regeneration [66]. The positive effect of HA in the context of periodontitis is attributable to a number of factors, including its angiogenesis and osteoinduction properties [67,68,69]. In vitro studies have demonstrated that HA enhances the osteogenic potential of bone morphogenetic protein-2 by inhibiting its antagonists [70,71,72]. In vivo research has shown that HA application enhances extraction socket healing with an increased expression of osteogenic proteins in rats [73,74].

The use of HA in conjunction with SRP protocols offers a promising approach to improving outcomes in periodontal treatment. Clinicians may consider HA for patients with deep periodontal pockets or refractory periodontitis, particularly given its regenerative potential and localized anti-inflammatory effects. However, the optimal clinical outcomes that can be achieved with the use of HA require the establishment of standardized protocols regarding the concentration of HA, the frequency of its application, and the duration of treatment. Furthermore, patient education on proper application techniques, especially for at-home use, is crucial to maximizing benefits. In addition, it has been demonstrated that patients who are informed about the natural origin of products exhibit higher levels of compliance [75,76].

Nevertheless, when considering the results in their entirety, it is imperative to acknowledge the existence of several inherent limitations. The most significant limitation is the quality of the resulting systematic reviews. As demonstrated by AMSTAR-2, all the articles included were of low or critically low quality. The authors of the source studies employed disparate concentrations of hyaluronic acid in their patients, as well as varying numbers of product applications. Furthermore, the disparate follow-up periods must be considered. One of the systematic reviews included studies with a follow-up period of only one week. A follow-up period of only one week is insufficient for inclusion in the analysis. A follow-up period of at least three months is required to evaluate the results. A further limitation that is likely to compromise the perception of the summary results presented by the authors is the inhomogeneity associated with the different methods of HA application itself. Some of the included systematic reviews included both subgingival and supragingival applications without separating their results, and it is, therefore, recommended that a future study compares different protocols for the application itself. Because of the aforementioned factors, the results presented exhibit a considerable degree of heterogeneity. Consequently, the authors of this umbrella review were unable to undertake any further statistical synthesis. Another limitation is the dearth of data among the included studies, which impairs the clarity and transparency of the articles themselves.

## 5. Conclusions

The adjunctive use of hyaluronic acid in SRP procedures shows potential for enhancing clinical outcomes in the non-surgical management of periodontitis. However, the quality of existing systematic reviews is variable, with significant heterogeneity in HA application protocols, follow-up durations, and study methodologies. Despite promising results in specific clinical parameters, these variations limit the strength of the conclusions drawn. Future high-quality research is essential to establish consistent protocols for HA application, concentration, and duration in periodontal therapy, ensuring reliable and standardized improvements in periodontal health.

## Figures and Tables

**Table 1:** Search terms used for specific databases with restrictions and the number of results.

Source	Search Term	Filters	Number of Results
Medline via PubMed	(“Periodontitis” OR “Periodontal Debridement” OR “Dental Scaling” OR “Root Planing” OR “Planing, Root” OR “Planings, Root” OR “Root Planings” OR “Subgingival Scaling” OR “Scaling, Subgingival” OR “Scalings, Root” OR “Root Scalings” OR “Debridement, Periodontal” OR “Debridements, Periodontal” OR “Periodontal Debridements” OR “Nonsurgical Periodontal Debridement” OR “Debridement, Nonsurgical Periodontal” OR “Debridements, Nonsurgical Periodontal” OR “Nonsurgical Periodontal Debridements” OR “Periodontal Debridement, Nonsurgical” OR “Periodontal Debridements, Nonsurgical” OR “Periodontal Pocket Debridement” OR “Debridement, Periodontal Pocket” OR “Debridements, Periodontal Pocket” OR “Periodontal Pocket Debridements” OR “Subgingival Curettage” OR “Curettages, Subgingival” OR “Curettage, Subgingival” OR “Subgingival Curettages” OR “Gingival Curettage” OR “Curettage, Gingival” OR “Curettages, Gingival” OR “Gingival Curettages” OR “Periodontal Epithelial Debridement” OR “Debridement, Periodontal Epithelial” OR “Debridements, Periodontal Epithelial” OR “Epithelial Debridement, Periodontal” OR “Epithelial Debridements, Periodontal” OR “Periodontal Epithelial Debridements”) AND (“Hyaluronic Acid” OR “Acid, Hyaluronic” OR “Hyaluronan” OR “Sodium Hyaluronate” OR “Hyaluronate, Sodium” OR “Hyaluronate Sodium”)	Systematic Review	8
Web of Science Scopus	(“Periodontitis” OR “Periodontal Debridement” OR “Dental Scaling” OR “Root Planing” OR “Planing, Root” OR “Planings, Root” OR “Root Planings” OR “Subgingival Scaling” OR “Scaling, Subgingival” OR “Scalings, Root” OR “Root Scalings” OR “Debridement, Periodontal” OR “Debridements, Periodontal” OR “Periodontal Debridements” OR “Nonsurgical Periodontal Debridement” OR “Debridement, Nonsurgical Periodontal” OR “Debridements, Nonsurgical Periodontal” OR “Nonsurgical Periodontal Debridements” OR “Periodontal Debridement, Nonsurgical” OR “Periodontal Debridements, Nonsurgical” OR “Periodontal Pocket Debridement” OR “Debridement, Periodontal Pocket” OR “Debridements, Periodontal Pocket” OR “Periodontal Pocket Debridements” OR “Subgingival Curettage” OR “Curettages, Subgingival” OR “Curettage, Subgingival” OR “Subgingival Curettages” OR “Gingival Curettage” OR “Curettage, Gingival” OR “Curettages, Gingival” OR “Gingival Curettages” OR “Periodontal Epithelial Debridement” OR “Debridement, Periodontal Epithelial” OR “Debridements, Periodontal Epithelial” OR “Epithelial Debridement, Periodontal” OR “Epithelial Debridements, Periodontal” OR “Periodontal Epithelial Debridements”) AND (“Hyaluronic Acid” OR “Acid, Hyaluronic” OR “Hyaluronan” OR “Sodium Hyaluronate” OR “Hyaluronate, Sodium” OR “Hyaluronate Sodium”)	Review	57
Embase	(‘Periodontitis’ OR ‘Periodontal Debridement’ OR ‘Dental Scaling’ OR ‘Root Planing’ OR ‘Planing, Root’ OR ‘Planings, Root’ OR ‘Root Planings’ OR ‘Subgingival Scaling’ OR ‘Scaling, Subgingival’ OR ‘Scalings, Root’ OR ‘Root Scalings’ OR ‘Debridement, Periodontal’ OR ‘Debridements, Periodontal’ OR ‘Periodontal Debridements’ OR ‘Nonsurgical Periodontal Debridement’ OR ‘Debridement, Nonsurgical Periodontal’ OR ‘Debridements, Nonsurgical Periodontal’ OR ‘Nonsurgical Periodontal Debridements’ OR ‘Periodontal Debridement, Nonsurgical’ OR ‘Periodontal Debridements, Nonsurgical’ OR ‘Periodontal Pocket Debridement’ OR ‘Debridement, Periodontal Pocket’ OR ‘Debridements, Periodontal Pocket’ OR ‘Periodontal Pocket Debridements’ OR ‘Subgingival Curettage’ OR ‘Curettages, Subgingival’ OR ‘Curettage, Subgingival’ OR ‘Subgingival Curettages’ OR ‘Gingival Curettage’ OR ‘Curettage, Gingival’ OR ‘Curettages, Gingival’ OR ‘Gingival Curettages’ OR ‘Periodontal Epithelial Debridement’ OR ‘Debridement, Periodontal Epithelial’ OR ‘Debridements, Periodontal Epithelial’ OR ‘Epithelial Debridement, Periodontal’ OR ‘Epithelial Debridements, Periodontal’ OR ‘Periodontal Epithelial Debridements’) AND (‘Hyaluronic Acid’ OR ‘Acid, Hyaluronic’ OR ‘Hyaluronan’ OR ‘Sodium Hyaluronate’ OR ‘Hyaluronate, Sodium’ OR ‘Hyaluronate Sodium’)	Systematic review Meta analysis	17
Google Scholar	(“periodontitis” or “SRP” or “root planing”) and (“hyaluronic acid”)	Systematic review	142

**Table 2:** Selection criteria for papers included in the umbrella review.

Inclusion Criteria	Exclusion Criteria
Systematic reviews Systematic reviews with meta-analysis English language Full text available Reviews based on human studies Non-surgical treatment of periodontitis	Case reports/Case series Narrative reviews Non-English language publications Letters to Editor Animal studies Conference papers Controlled trials Only the surgical treatment of periodontitis was included in the reviews

**Table 3:** Evaluation of parameters included in AMSTAR-2.

Author/Year	Q1	Q2	Q3	Q4	Q5	Q6	Q7	Q8	Q9	Q10	Q11	Q12	Q13	Q14	Q15	Q16	Review Quality
Alshehri and Alharbi (2023) [39]	Y	Y	N	PY	Y	Y	Y	PY	PY	N	Y	N	N	Y	N	Y	Critically low
Dharmadhikari (2024) [40]	Y	Y	N	PY	Y	Y	N	PY	Y	N	Y	Y	N	Y	Y	N	Low
Eliezer et al. (2019) [41]	Y	PY	N	Y	Y	Y	Y	PY	Y	Y	Y	N	N	Y	Y	Y	Low
Karakostas et al. (2022) [42]	Y	PY	N	PY	Y	N	N	PY	Y	Y	N/A	N/A	Y	Y	N/A	Y	Low
Rodrigues et al. (2020) [43]	Y	Y	N	PY	Y	Y	Y	N	PY	Y	N/A	N/A	N	N	N/A	Y	Low

N—No, PY—Partial Yes, Y—Yes, N/A—Not Applicable, Q1—Did the research questions and inclusion criteria for the review include the components of PICO? Q2—Did the report of the review contain an explicit statement that the review methods were established before the conduct of the review, and did the report justify any significant deviations from the protocol? Q3—Did the review authors explain their selection of the study designs for inclusion in the review? Q4—Did the review authors use a comprehensive literature search strategy? Q5—Did the review authors perform study selection in duplicate? Q6—Did the review authors perform data extraction in duplicate? Q7—Did the review authors provide a list of excluded studies and justify the exclusions? Q8—Did the review authors describe the included studies in adequate detail? Q9—Did the review authors use a satisfactory technique for assessing the risk of bias (RoB) in individual studies that were included in the review? Q10—Did the review authors report on the sources of funding for the studies included in the review? Q11—If meta-analysis was performed, did the review authors use appropriate methods for the statistical combination of results? Q12—If meta-analysis was performed, did the review authors assess the potential impact of RoB in individual studies on the results of the meta-analysis or other evidence synthesis? Q13—Did the review authors account for RoB in primary studies when interpreting/discussing the results of the review? Q14—Did the review authors provide a satisfactory explanation for, and discussion of, any heterogeneity observed in the results of the review? Q15—If they performed quantitative synthesis, did the review authors carry out an adequate investigation of publication bias (small study bias) and discuss its likely impact on the results of the review? Q16—Did the review authors report any potential sources of conflict of interest, including any funding they received for conducting the review?

**Table 4:** Guidelines for assessing the quality of the review based on the answers to each question.

Quality of the Review	Criteria
High	Zero or one non-critical weakness: The systematic review provides an accurate and comprehensive summary of the results of the available studies that address the question of interest.
Moderate	More than one non-critical weakness: The systematic review has more than one weakness but no critical flaws. It may provide an accurate summary of the results of the available studies that were included in the review.
Low	One critical flaw with or without non-critical weaknesses: The review has a critical flaw and may not provide an accurate and comprehensive summary of the available studies that address the question of interest.
Critically low	More than one critical flaw with or without non-critical weaknesses: The review has more than one critical flaw and should not be relied on to provide an accurate and comprehensive summary of the available studies.

**Table 5:** Characteristics included systematic reviews.

Author/Year	Number of Patients	Number of Sites	Age (Years) (Mean)	Gender	Search Period	Tool Used for Quality Assessment
Alshehri and Alharbi (2023) [39]	113	No data	51.7	38.5% males 61.5% females	Up to 25 September 2022	Not mentioned
Dharmadhikari (2024) [40]	579	No data	No data	No data	Up to November 2023	Newcastle Ottawa Scale, ROB-2
Eliezer et al. (2019) [41]	277	No data	No data	No data	No data	ROB-2
Karakostas et al. (2022) [42]	494	No data	No data	No data	Up to February 2021	ROB-2
Rodrigues et al. (2020) [43]	No data	No data	No data	No data	Up to 18 May 2018	ROB-2

ROB-2—Cochrane risk of bias assessment tool for randomized trials.

**Table 6:** Detailed characteristics of included studies.

Author/Year	Number of Included Studies	Study Types	HA Concentration Range	Number of Sessions of HA Application	Methods of Analysis	Follow-Up Range	Conclusions
Alshehri and Alharbi (2023) [39]	5	Randomized (4) and non-randomized (1) controlled trials	0.2–1.4%	3–29	SR/MA	3–6 months	The use of HA as an adjunct to NSPT did not result in a discernible additional benefit with respect to the reduction in the prevalence of *Porphyromonas gingivalis* in subgingival biofilms.
Dharmadhikari (2024) [40]	17	Clinical studies, comparative studies, prospective studies, retrospective studies	0.2–0.8%	No data	SR/MA	No data	The subgingival application of HA was found to result in a notable and advantageous improvement in periodontal parameters. It was determined that HA can be utilized as a standalone agent or in conjunction with NSPT/SRP for the enhancement of periodontal health.
Eliezer et al. (2019) [41]	11	Randomized controlled trial	0.2–0.8%	1–4	SR/MA	3–6 months	The available evidence suggests that the topical application of HA may result in enhanced clinical outcomes when used in conjunction with non-surgical periodontal therapy.
Karakostas et al. (2022) [42]	20	Randomized controlled trial	0.2–0.8%	1–7	SR	1 week–1 year	The adjunctive use of HA may result in a reduction in the prescription of nonsteroidal anti-inflammatory drugs and an improvement in clinical parameters, including periodontal probing depth, periodontal inflammation, and clinical attachment level.
Rodrigues et al. (2020) [43]	7	Randomized controlled trial	0.2–0.8%	No data	SR	12 months	The combination of HA and SRP yields superior outcomes in the majority of outcome variables when compared to the conventional treatment of SRP alone in the context of periodontitis. The use of HA as an adjuvant in periodontal therapy is a viable option that merits consideration.

HA—Hyaluronic Acid, SR—Systematic Review, SR/MA—Systematic Review with Meta-analysis, SRP—Scaling and Root Planing, NSPT—Non-surgical Periodontal Therapy.

## Data Availability

No new data were created or analyzed in this study. Data sharing is not applicable.
